# Diagnostic Test to Identify Parkinson's Disease from the Blood Sera of Chinese Population: A Cross-Sectional Study

**DOI:** 10.1155/2022/8683877

**Published:** 2022-04-06

**Authors:** Guangan Tong, Pingping Zhang, Wenbin Hu, Kun Zhang, Xianwen Chen

**Affiliations:** ^1^Department of Neurology, The First Affiliated Hospital of Anhui Medical University, Hefei 230031, Anhui, China; ^2^The Affiliated Hospital of the Neurology Institute, Anhui University of Chinese Medicine, Hefei 230031, Anhui, China; ^3^Department of Physiology, School of Basic Medicine, Anhui Medical University, Hefei 230012, Anhui, China; ^4^Department of Neurology, People's Hospital of Taihe County, Taihe 236600, Anhui, China

## Abstract

**Background:**

Parkinson's disease (PD) is a neurodegenerative disease, a hallmark by the formation of misfolded and aggregated *α*-synuclein proteins. The expression of potential microRNA (miRNA) candidates isolated from serum and cerebrospinal fluid (CSF) exosomes of PD patients was assessed for their diagnostic value and their potential role as biomarkers for PD was explored. In this study, we characterize the expression level of miRNAs in the exosomes of blood sera and cerebrospinal fluid and explore their potential role as biomarkers for PD.

**Materials and Methods:**

A total of 209 patients having an onset of PD, along with 60 neurodegenerative (ND) disorders and 50 healthy controls were enrolled. Blood samples and CSF samples were collected and exosomes were isolated. The isolated exosomes were characterized using CD63 detection and exosomal RNA was extracted. Serum miRNA profiling was carried out by synthesizing cDNA from the purified RNA and miRNA transcripts were determined by qRT-PCR using SYBR Green PremixScript. microRNA profiling strategy was employed for extracting the exosomal miRNAs from the exosomes.

**Results:**

Five common miRNAs viz. miR-151a-5p, miR-24, mir-485-5p, mir-331-5p, and mir-214 were found to be upregulated with statistical significance in both the serum exosome and CSF exosomes. The investigation revealed that serum and CSF exosomal miRNA molecules are definitive biomarkers for PD with proper specificity and sensitivity.

**Conclusions:**

The significant level of miR-151a-5p, miR-24, mir-485-5p, mir-331-5p, and mir-214 was observed in the serum and CSF which may be established as a biomarker for the diagnosis of PD.

## 1. Introduction

Parkinson's disease (PD) is a neurodegenerative disorder that affects nearly 10 million individuals globally [1]. It completely affects the life standard and living of an individual and becomes dependent or requires a helper, thereby leading to premature mortality [2]. The symptoms of PD generally include motor symptoms characterized by bradykinesia, tremor, postural instability, and rigidity. The nonmotor symptoms of PD include cognitive impairment, anxiety, and depression [3]. In China, it is estimated that the total number of PD cases will be increased to 5 million by 2030, which accounts for half of the global cases [1, 2]. It is approximated around 25% of the Chinese population will cross 65 years by 2050 and is estimated to impact the increasing aged population [4]. In spite of PD being a motor disease showing disability, various reports observed symptoms prior to motor symptoms, revealing the onset of the disease pathology years ahead of its distinctive symptoms [5]. Moreover, the worst part is that 25% of the individual's neurons in the substantia nigra and striatum fibers are damaged when the motor symptoms show up at the time of diagnosis [6]. To date, there is no specific and accurate test for the concrete diagnosis of PD and most of the current diagnostics rely on the patient's medical evaluation, neuropsychological assessments, clinical criteria, brain imaging such as computerized tomography (CT) scan, and magnetic resonance imaging (MRI) [7, 8]. This is because the clinical symptoms of PD in its early stage are very similar to Alzheimer's disease, dementia, sclerosis, and other neurodegenerative diseases [9]. Thus, an accurate diagnosis is sometimes difficult in its early stages and there is a high chance of wrong diagnosis [10]. Therefore, there is an urgent need for reliable biomarkers for proper treatment and disease progression of PD. It is reported that microRNA (miRNA), which is usually packed in exosomes, are involved in the pathogenesis of PD and miRNAs play a key process in the upregulation of certain proteins which is related to PD such as *α*-synuclein, tau protein, DJ-1, etc [11, 12]. miRNAs and exosomes form an association that works in a synergetic way and is involved in the pathogenesis of PD [13]. miRNAs are also thought to play a key role in the pathogenesis pathway of PD, which includes protein aggregation, autophagy, and inflammation [14]. Therefore, miRNA is considered a potent class of promising biomarkers for PD. Extracellular miRNAs are usually enclosed in exosomal microvesicles secreted out into body fluids such as blood, plasma, and cerebrospinal fluid (CSF) [15]. miRNA profiles in exosomes are distinct in PD compared to other neurodegenerative diseases, which makes it an efficient prognostic marker for the disease progression [16]. Because of the potent role of miRNA in the upregulation of proteins associated with Parkinson's disease and its association with the pathogenesis of Parkinson's disease, miRNA is appraised as a convincing biomarker for the diagnosis of PD. Therefore, the present investigation aims to characterize the expression level of miRNAs in the exosomes of blood sera and cerebrospinal fluid (CSF) of PD patients and to explore the potential role of miRNA as biomarkers for PD.

## 2. Materials and Methods

### 2.1. Ethical Approval and Statement

All experiments were carried out in accordance with the Declaration of Helsinki and approved by the Medical Ethics Committee of the Hospital (Approval No. AKT/091826912-A19 dated 5 Feb 2018). Written informed consent was obtained from all participants. The enrolled patients were free from severe medical conditions such as vascular diseases, hypertension, diabetes, and liver diseases.

### 2.2. Participants and Patient's Samples

209 patients having an onset of PD were enrolled in the hospital between June 2018–July 2021 and were included in the study. All patients were investigated in the Neurology and Psychiatric Dept. of the Hospital. The PD patients were further evaluated for cognitive function and their behavioral and psychological symptoms were observed. Additional 60 neurodegenerative disorder (ND) patients (20 Alzheimer's disease (AD), 20 multiple sclerosis (MS), and 20 Huntington's disease (HD)) and another 50 healthy control participants were also enrolled from our institute who had no episodes of cognitive impairment or neurological disorder. The healthy control was also evaluated with similar cognitive examinations and brain CT scans. The baseline characteristics and demographic details of the participants were recorded and the onset of PD was characterized and evaluated.

### 2.3. Serum Samples

Around 5 mL of blood samples were collected from 209 PD patients, 60 ND patients, and 50 healthy controls. Blood samples were collected through vein puncture using serum clot activator tubes. The blood samples were processed based on the standard operating procedure and specific guidelines of our institute. The blood samples were kept in a standing position for 15 min at 5°C and the serum was isolated by centrifugation at 3000 × *g* for 20 min at 28°C. The serum samples were further preserved in a deep fridge (−80°C) for further analysis.

### 2.4. CSF Samples

CSF samples were collected by puncturing the lumbar in polypropylene tubes and centrifuged at 1500 × *g* for 12 min. The CSF aliquots were then preserved at −80 C until further analysis. The samples were then analyzed for T-tau, Alpha-synuclein, and DJ-1 protein levels. The protein levels were determined and evaluated using a sandwich ELISA and Luminex assay.

### 2.5. Exome Isolation

Exosomes were extracted from the serum and CSF samples using ExoQuick-TC (SBI, California, United States) following the manufacturer's protocol. The samples were centrifuged at 2,000 × *g* for 20 min followed by additional centrifugation at 7,500 × *g* for 8 min to remove the cell debris. The supernatants were then filtered using a filter membrane and around 500 *μ*L of the debris was mixed with 0.3 volumes of the reagent and kept for incubation for 40 min at 6°C. The reagent mixed samples were further centrifuged at 9,000 × *g* for 12 min and the resultant pellets were treated with phosphate-buffered saline (PBS) solution. It was further centrifuged again, and re-suspended in prefiltrated PBS solution for the isolation of ribonucleic acid (RNA). Exosome preparations were also stored at −80°C for future use or until analysis.

### 2.6. Characterization of Serum Exosomes and CSF Exosomes

The isolated serum exosomes were characterized using a CD63 Detection reagent (Thermo) and further stained with a CD63 monoclonal antibody (Thermo) for the analysis of flow cytometry.

CSF exosomes were characterized using the fluorescence-activated cell sorting(FACS) technique. The CSF samples were captured on Dynabeads and characterized using the CD63 monoclonal antibody. The beads were fluorescence-labeled with their respective anti-CD63, anti-CD86, and anti-CD54 controls and analyzed using BD FACS Calibur (BD Biosciences).

### 2.7. Isolation of RNA from Exosomes

Exosomal RNA extraction was carried out from the supernatants using an EZ RNA Miniprep Kit (EZ BioResearch, Missouri, United States) following the standard protocol. The samples were mixed properly and kept in a shaker for 2 min with uniform shaking and incubated at 34 C for 15 min. The samples were then centrifuged at 10000 × *g* for 10 min at 4C for separating the upper aqueous phase. The resultant RNA pellets were then washed with a RPE buffer and eluted in nuclease-free water. The purified RNA was quantified using the Nanodrop ND-1000 (Thermo Fischer Scientific) and analyzed using real-time polymerase chain reaction (PCR) and quantitative polymerase chain reaction (qPCR).

### 2.8. Profiling of miRNA and TaqMan Assay

Serum miRNA profiling was carried out by synthesizing the complementary deoxyribonucleic acid(cDNA) from the purified RNA using a PrimeScript miRNA Standard Kit (United States) based on the standard protocol and cycling conditions. The reverse transcription was initiated by adding a poly-A tail to the 3' end and the process was followed by ligation with oligo-dT at the 5' end of the miRNA. CFS global miRNA profiling was carried out using TaqMan Low-Density Array Human miRNA Panels (Applied Biosystems, Foster City, California) by synthesizing the cDNA following the manufacturer's protocol and cycling conditions. The reverse transcription was initiated by quantitative PCR performed on an Applied Biosystems 7900HT thermocycler (Applied Biosystems). Furthermore, the exosome samples from the serum and CSF were analyzed using quantitative PCR and quantified by TaqMan miRNA assays (Applied Biosystems, Foster City, California) for the differential expression of miRNAs from the array panel.

### 2.9. RT-qPCR Analysis

The miRNA transcripts were determined by quantitative real-time PCR using the SYBR Green PremixScript miRNA real-time PCR kit (Applied Biosystem). The real-time PCR was carried out using an ABI thermocycler (Applied Biosystems, Life, United States) using standard PCR conditions. All reactions were assayed and carried out in triplicate. The expression level of the miRNA was quantified by ΔCt by fixing at 0.05 unit of basal fluorescence and taking *C*(*t*) control as the reference value.

### 2.10. Statistical Analysis

Statistical analysis was carried out using SPSS 17.0 (Chicago, Illinois, United States). Student *t*-test was used for differentiating between the groups and *P* < 0.05 was taken for statistical significance. Receiver operation characteristic curves (ROC curve) were utilized to evaluate the sensitivity and specificity of miRNAs for possible diagnostic markers for Parkinson's disease.

## 3. Results

**Figure 1:**
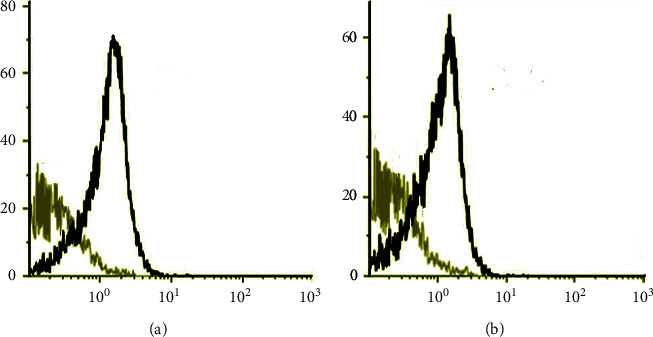
Flow cytometry analysis of (a) serum and (b) cerebrospinal fluid (CSF) exosomes displayed expression of CD63 surface markers.

The demographic characteristics of the patients are presented in Table 1. The mean age of the PD group was 68.2 ± 5.42 years compared to 69.4 ± 7.1 years of ND group and 65.6 ± 4.3 years of the healthy control group (Table 1). There were no significant differences based on the smoking status and alcohol consumption among the three groups. The level of a-synuclein in the serum and CSF in the PD and ND groups was comparatively higher compared to the healthy group. Similarly, the level of tau protein in the CSF was comparatively higher in the PD and ND groups (Table 1). The presence of surface protein CD63 was found to be present in both the serum and the CSF exosome of the PD patients from the flow cytometry analysis (Figures 1(a) and 1(b)).

CD63 protein is considered a common marker for exosomes. Furthermore, the serum and CSF-derived exosomes were verified for the presence of RNA. The exosomes were treated with RNAase solution mixed with 2% Triton and the degraded exosomal RNA was confirmed by running the gel using electrophoresis technique. The expression of exosomal miRNA in serum samples and CSF samples of PD patients were profiled based on TaqMan miRNA arrays, which comprise more than 900 miRNAs. The relative abundance of exosomal miRNAs was detected by normalizing the samples with the microRNA gene card RNU6-B. A total of 27 exosomal miRNAs showed differential expression in serum samples as well as in the CSF samples from PD patients. In the case of serum samples, 17 exosomal miRNAs were upregulated and 10 miRNAs were underregulated with *P* < 0.05 compared to the healthy control. Whereas, in CSF samples, 19 exosomal miRNAs were upregulated and 8 miRNAs were underregulated with *P* < 0.05 compared to the healthy control (Table 2). The expression levels of miR-24, miR-126a, miR-301a, and miR-16-2-3p were comparatively high in the PD serum samples compared with the control group ((*P* < 0.05)), whereas the levels of miR-29b-2-5p and let-7d-5p were remarkably lower in the PD serum samples compared to healthy controls (*P* < 0.05). Similarly, the expression levels of miR-200a-5p, mir-22, mir-214, and mir-22 were significantly higher in CSF samples from patients with PD compared with the control group ((*P* < 0.05)) and the levels of mir-26a and mir-409-3p were significantly lower in CSF samples compared to controls (*P* < 0.05).

**Figure 2:**
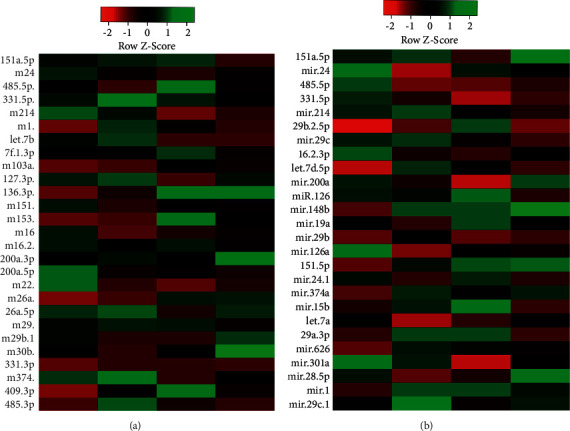
Heat map plot of (a) serum and (b) cerebrospinal fluid (CSF) microRNA (miRNA).

Interestingly, miRNA viz. miR-151a-5p, miR-24, mir-485-5p, mir-331-5p, and mir-214 were expressed in both serum exosomes and CSF exosomes. These results suggest that these miRNAs may be the key miRNA for the early diagnosis of PD. These data revealed that miRNAs were present in serum and CSF exosomes. Moreover, it was confirmed that the exosomal miRNAs were differentially expressed in the serum and CSF samples of PD patients compared to healthy controls (Table 2). For the validation of the exosome samples, Taqman RT-PCR was employed to validate the expression level 5 miRNA, which is expressed in both the serum samples and CSF samples from the miRNA assay. These miRNAs had a good statistical significance which included the *p*-value and fold change compared to the healthy control and ND (Table 2). The heatmap analysis of the differential miRNA signature of the serum and CSF samples representing the grid matrix of exosomal miRNAs are shown in Figure 2(a) (serum) and Figure 2(b) (CSF).

**Figure 3:**
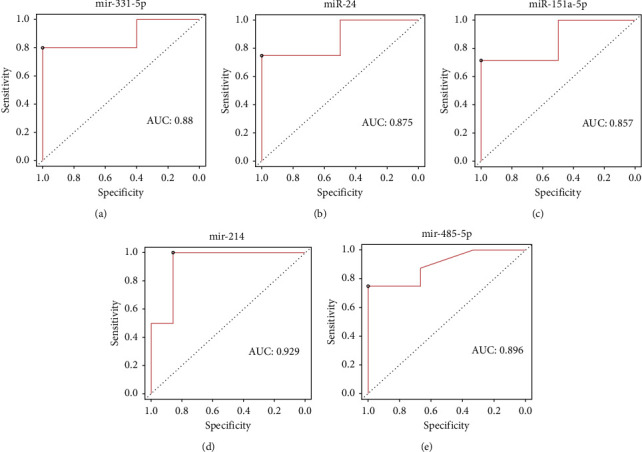
Receiver operation characteristic curve (ROC curve) of the microRNA (miRNA) expressed in serum and cerebrospinal fluid (CSF) exosomes which is significant in Parkinson's disease (PD) compared to neurodegenerative disorder (ND) and control.

The Taqman assay observed that all miRNA viz miR-151a-5p, miR-24, mir-485-5p, mir-331-5p, and mir-214 were significantly overexpressed in the serum and the CSF exosome samples. Furthermore, the utility of serum miRNA and CSF miRNA was evaluated using ROC curves to distinguish their predictive abilities. The ROC results observed that the five common miRNAs from serum and CSF, which were differentially expressed, had a significant diagnostic ROC value for PD compared to healthy controls (Figure 3). The area under the curve (AUC) values were miR-151a-5p, AUC = 0.857 (95% confidence interval, CI, 0.835–0.901); miR-24, AUC = 0.875 (95% CI, 0.754–0.853); mir-485-5p, AUC = 0.896 (95% CI, 0.753–0.842); mir-331-5p, AUC = 0.88 (95% CI, 0.912–0.749); and mir-214, AUC = 0.929 (95% CI, 0.843–0.921). The sensitivity and specificity at the optimal cutoff value for differentiating PD were 89.23% for miR-151a-5p, 92.35% for miR-24, 95.34% for mir-485-5p, 85.34% for mir-331-5p, and 82.34% for mir-214. These AUC results suggest that these five miRNAs viz. miR-151a-5p, miR-24, 485-5p, mir-331-5p, and mir-214 had a good diagnostic value in PD. Therefore, these data revealed that exosomal miRNAs in serum and CSF are reliable diagnostic markers for PD.

## 4. Discussion

The histopathological characterization of PD is mainly determined by the formation of the Lewy bodies and the presence of *α*-syn. The progression of PD is not fully understood; however, the presence of *α*-syn is believed to play a crucial role in the disease progression. Exosomes are also thought to play a crucial role in the progression of PD [17]. On the other hand, the progression of PD is mostly characterized by symptoms such as dementia, tremor, and postural instability. However, there is no accurate test or diagnostics test for PD, and most of the diagnostic for PD is based on its clinical symptoms [18]. In this study, we have, for the first time, verified the presence of five common miRNAs (miR-151a-5p, miR-24, mir-485-5p, mir-331-5p, and mir-214) in both the serum exosomes and CSF exosomes of PD patients. This finding suggests that these miRNAs could be important for the early diagnosis of PD. However, there were individual reports on the presence of miR-151a-5p and miR-24 in blood [19, 20] and CSF samples [21, 22]. Moreover, a miRNA profile was also substantially expressed in the serum and CSF samples of PD patients. The miR-24 and mir-485-5p were found to be downregulated in the ND group compared to the serum and CSF samples. These results also help in identifying the differentiation between PD and other ND such as Alzheimer's disease, Huntington's disease, etc. There are studies in the past that focus on the identification of a specific miRNA in PD, which results in revealing the functional relevance of miRNA to the onset and disease progression of PD [23, 24]. The earliest study on miRNAs in PD dates to 2007 conducted by Kim et al. where the workers evaluated 230 panels of miRNA precursors from the brain [25]. The study also observed that the neurotrophin signaling pathway was prominent with various exosomal miRNA patterns in the serum and CSF samples of PD patients. The presence of exosomes is a perfect approach to identify the function of miRNAs in the progression and pathogenesis of Parkinson's disease. Exosomal surface proteins such as CD63 and CD81 are commonly used for identifying the content of exosomes [26]. In our case, the CD63 protein was detected in both the samples from the serum and CSF using the FACS technique. In fact, molecular biomarkers could be used as a potential diagnostic test for PD because there are no accurate diagnostic tests for PD to date. There are also other potent biomarkers for PD such as detection of key proteins which include *α*-synuclein, tau protein, and DJ-1 in the CSF and brain tissue [20, 27]. Furthermore, the RT-qPCR analysis confirmed that 5 common miRNAs (miR-151a-5p, miR-24, mir-485-5p, mir-331-5p, and mir-214) in serum exosomes and CSF exosomes of PD patients were upregulated when compared to the healthy control group and the ND group.

The use of RT-qPCR is regular and is widely for the analysis of miRNA because this technique is considered as a gold standard with high sensitivity and specificity [28]. The RT-qPCR technique also has advantages in providing fast and high-throughput detection with immediate information [29]. Thus, these five biomarkers may demonstrate a very high predictive biomarker performance. However, it may require additional research to point out the biological function and role of these miRNAs in the onset of PD.

## 5. Conclusion

In conclusion, the detection of miR-151a-5p, miR-24, mir-485-5p, mir-331-5p, and mir-214 in the serum exosome and CSF exosomes may provide substantial aid in the early diagnosis of PD. However, other potent miRNAs may also establish a significant relationship with PD and this could not be observed in the present study because of the limited sample size. Therefore, the present study concludes that the significant level of miR-151a-5p, miR-24, mir-485-5p, mir-331-5p, and mir-214 in the serum and CSF may be established as a biomarker for the diagnosis of PD.

## Figures and Tables

**Table 1:** Demographic characteristics of Parkinson's disease (PD), neurodegenerative disorder (ND), and control subjects.

Characteristics	PD (*n* = 209)	Healthy control (*n* = 50)	ND (*n* = 60)
Age (years)	68.2 ± 5.42	65.6 ± 4.3	69.4 ± 7.1
Male	115 (54.02%)	29 (58.0%)	35 (58.33%)
Female	94 (44.98%)	21 (42.0%)	25 (41.67%)
Cigarette smoker	63 (30.14%)	15 (30.0%)	19 (31.67%)
Nonsmoker	146 (69.86%)	35 (70.0%)	41 (68.33%)
Alcohol drinker	81 (38.76%)	23 (46.0%)	26 (43.33%)
Nondrinker	128 (61.24%)	27 (54.0%)	34 (56.67%)
*α*-Synuclein in CSF (pg/*μ*l)	1.36 ± 0.34	0.69 ± 0.48	1.76 ± 0.31
*α*-Synuclein in serum (pg/*μ*l)	0.06 ± 0.51	0.01 ± 0.02	0.08 ± 0.12
Tau protein in CSF (pg/ml)	219 ± 43	116 ± 32	421 ± 93

CFS = cerebrospinal fluid.

**Table 2:** Differential exosomal microRNA (miRNA) expression in serum and cerebrospinal fluid (CSF) samples of Parkinson's disease (PD), neurodegenerative disorder (ND), and control subjects.

miRNA member	Serum				miRNA member	CSF			
	PD VS control		PD VS ND			PD VS control		PD VS ND	
hsa-mirName	FC (log2)	Adjusted *p* value	FC (log2)	Adjusted *p* value	has-mirName	FC (log2)	Adjusted *p* value	Fold change (log2)	Adjusted *p* value
	Up + 17	Dn- = 10				Up + 19	Dn- = 8		
hsa-miR-151a-5p	1.37	0.0042	0.74	0.313	hsa-miR-151a-5p	1.31	0.0073	0.51	0.035
hsa-miR-24	2.31	0.0074	−0.96	0.131	hsa-miR-24	1.62	0.0028	−0.19	0.173
hsa-miR-485-5p	1.95	0.0032	−0.78	0.061	hsa-mir-485-5p	1.79	0.0087	−0.56	0.169
hsa-miR-331-5p	1.63	0.0024	−0.27	0.042	hsa-mir-331-5p	1.43	0.0067	1.34	0.231
hsa-mir-214	1.45	0.0064	0.85	0.067	hsa-mir-214	2.18	0.0013	0.37	0.052
hsa-miR-29b-2-5p	−1.56	0.0086	−0.63	0.008	hsa-mir-1	−0.75	0.0043	0.63	0.042
hsa-miR-29c	1.43	0.0053	0.74	0.045	hsa-let-7b	1.23	0.0023	0.72	0.013
hsa-miR-16-2-3p	2.04	0.0042	−0.24	0.141	hsa-let-7f-1-3p	0.84	0.0075	0.13	0.143
hsa-let-7d-5p	−1.42	0.0065	0.68	0.042	hsa-mir-103a	−0.64	0.0054	−0.63	0.125
hsa-miR-200a	1.43	0.0021	−0.24	0.214	hsa-mir-127-3p	1.62	0.0022	0.76	0.315
hsa-miR-126	1.39	0.0089	0.34	0.061	hsa-mir-136-3p	−0.63	0.0095	−0.28	0.531
hsa- miR-221	0. 91	0.0024	−0.15	0.162	hsa-mir-151	1.56	0.0044	−0.47	0.173
hsa- miR-148b	−0.76	0.0086	0.85	0.341	hsa-mir-153	−0.72	0.0093	−0.63	0.251
hsa- miR-19a	1.02	0.0086	−0.46	0.142	hsa-miR-16	1.53	0.0031	−0.71	0.152
hsa-miR-29b	−0.84	0.0032	0.09	0.041	hsa-mir-16-2	1.46	0.0064	0.08	0.173
hsa-miR-126a	2.46	0.0065	−0.85	0.085	hsa-miR-200a-3p	1.08	0.0046	0.38	0.663
hsa-miR-151-5p	−0.82	0.0087	0.47	0.231	hsa-miR-200a-5p	2.42	0.0043	−0.21	0.113
hsa- miR-24	1.18	0.0079	−0.41	0.062	hsa-mir-22	2.31	0.0015	−0.52	0.085
hsa-miR-374a	−0.73	0.0067	0.65	0.184	hsa-mir-26a	−1.35	0.0065	−0.63	0.352
hsa-miR-15b	−0.16	0.0096	0.54	0.042	hsa-miR-26a-5p	1.86	0.0032	0.82	0.374
hsa-let-7a	0.66	0.0041	−0.96	0.139	hsa-mir-29	1.24	0.0065	0.48	0.139
hsa-miR-29a-3p	−0.43	0.0085	0.85	0.045	hsa-miR-29b-1	1.21	0.0028	−0.46	0.421
hsa- miR-626	−0.84	0.0069	0.52	0.101	hsa-mir-30b	0.84	0.0054	−0.51	0.732
hsa-miR-301a	2.71	0.0022	0.55	0.126	hsa-miR-331-3p	−0.72	0.0025	−0.52	0.024
hsa-miR-28-5p	1.31	0.0046	−0.71	0.272	hsa-mir-374	2.04	0.0026	1.06	0.214
hsa-miR-1	−0.34	0.0085	0.87	0.168	hsa-mir-409-3p	−1.12	0.0086	−0.09	0.118
hsa- miR-29c	0.96	0.0053	1.39	0.172	hsa-miR-485-3p	−0.44	0.0034	0.87	0.031

VS = versus; FC = fold change; Dn Downregulated; hsa-miR = human miRNA; PD = Parkinson's disease; ND = neurodegenerative disease.

## Data Availability

The datasets in this study can be obtained from the corresponding author upon reasonable request.

## References

[1] Zheng B., Liao Z., Locascio J. J. (2010). PGC-1*α*, a potential therapeutic target for early intervention in Parkinson’s disease. *Science Translational Medicine*.

[2] Dinda B., Dinda M., Kulsi G., Chakraborty A., Dinda S. (2019). Therapeutic potentials of plant iridoids in alzheimer’s and parkinson’s diseases: a review. *European Journal of Medicinal Chemistry*.

[3] Balestrino R., Schapira A. H. V. (2020). Parkinson disease. *European Journal of Neurology*.

[4] Chen W., Xu Z.-M., Wang G., Chen S.-D. (2012). Non-motor symptoms of Parkinson’s disease in China: a review of the literature. *Parkinsonism & Related Disorders*.

[5] Amano S., Kegelmeyer D., Hong S. L. (2015). Rethinking energy in parkinsonian motor symptoms: a potential role for neural metabolic deficits. *Frontiers in Systems Neuroscience*.

[6] Earhart G. M., Falvo M. J. (2013). Parkinson disease and exercise. *Comprehensive Physiology*.

[7] Ozsahin I., Sekeroglu B., Pwavodi P. C., Mok G. S. P. (2020). High-accuracy automated diagnosis of parkinson’s disease. *Current Medical Imaging Formerly Current Medical Imaging Reviews*.

[8] Dissanayaka N. N. N. W., White E., O’Sullivan J. D., Marsh R., Pachana N. A., Byrne G. J. (2014). The clinical spectrum of anxiety in parkinson’s disease. *Movement Disorders*.

[9] Wu Y., Yao Q., Jiang G.-X., Wang G., Cheng Q. (2020). Identification of distinct blood-based biomarkers in early stage of parkinson’s disease. *Neurological Sciences*.

[10] Del Pino R., Díez-Cirarda M., Peña J., Ibarretxe-Bilbao N., Ojeda N. (2020). Estimation of cognitive performance based on premorbid intelligence in parkinson’s disease. *Journal of Parkinson’s Disease*.

[11] Yu H., Sun T., An J. (2020). Potential roles of exosomes in parkinson’s disease: from pathogenesis, diagnosis, and treatment to prognosis. *Frontiers in Cell and Developmental Biology*.

[12] Leggio L., Vivarelli S., L’Episcopo F. (2017). microRNAs in parkinson’s disease: from pathogenesis to novel diagnostic and therapeutic approaches. *International Journal of Molecular Sciences*.

[13] Li D., Li Y.-P., Li Y.-X. (2018). Effect of regulatory network of exosomes and microRNAs on neurodegenerative diseases. *Chinese Medical Journal*.

[14] Wu X., Meng X., Tan F. (2019). Regulatory mechanism of miR-543-3p on GLT-1 in a mouse model of parkinson’s disease. *ACS Chemical Neuroscience*.

[15] Pinnell J. R., Cui M., Tieu K. (2021). Exosomes in parkinson disease. *Journal of Neurochemistry*.

[16] Dos Santos M. C. T., Barreto-Sanz M. A., Correia B. R. S. (2018). miRNA-based signatures in cerebrospinal fluid as potential diagnostic tools for early stage parkinson’s disease. *Oncotarget*.

[17] Antony P. M. A., Diederich N. J., Krüger R., Balling R. (2013). The hallmarks of parkinson’s disease. *FEBS Journal*.

[18] Aaseth J., Dusek P., Roos P. M. (2018). Prevention of progression in parkinson’s disease. *Biometals*.

[19] Hoss A. G., Labadorf A., Beach T. G., Latourelle J. C., Myers R. H. (2016). microRNA profiles in parkinson’s disease prefrontal cortex. *Frontiers in Aging Neuroscience*.

[20] Gui Y., Liu H., Zhang L., Lv W., Hu X. (2015). Altered microRNA profiles in cerebrospinal fluid exosome in parkinson disease and alzheimer disease. *Oncotarget*.

[21] Chatterjee P., Roy D. (2017). Comparative analysis of RNA-Seq data from brain and blood samples of parkinson’s disease. *Biochemical and Biophysical Research Communications*.

[22] Marques T. M., Kuiperij H. B., Bruinsma I. B. (2017). MicroRNAs in cerebrospinal fluid as potential biomarkers for parkinson’s disease and multiple system atrophy. *Molecular Neurobiology*.

[23] Chen L., Yang J., Lü J., Cao S., Zhao Q., Yu Z. (2018). Identification of aberrant circulating miRNAs in parkinson’s disease plasma samples. *Brain and Behavior*.

[24] Yan J.-H., Hua P., Chen Y. (2020). Identification of microRNAs for the early diagnosis of parkinson’s disease and multiple system atrophy. *Journal of Integrative Neuroscience*.

[25] Kim J., Inoue K., Ishii J. (2007). A MicroRNA feedback circuit in midbrain dopamine neurons. *Science*.

[26] Picca A., Guerra F., Calvani R. (2020). Mitochondrial signatures in circulating extracellular vesicles of older adults with parkinson’s disease: results from the EXosomes in PArkiNson’s disease (EXPAND) study. *Journal of Clinical Medicine*.

[27] Parnetti L., Castrioto A., Chiasserini D. (2013). Cerebrospinal fluid biomarkers in parkinson disease. *Nature Reviews Neurology*.

[28] Mestdagh P., Van Vlierberghe P., De Weer A. (2009). A novel and universal method for microRNA RT-qPCR data normalization. *Genome Biology*.

[29] Siddika T., Heinemann I. U. (2021). Bringing MicroRNAs to Light. Methods for MicroRNA quantification and visualization in live cells. *Frontiers in Bioengineering and Biotechnology*.

